# Palladium-catalysed efficient synthesis of primary alkyl halides from terminal and internal alkenes

**DOI:** 10.1093/nsr/nwac198

**Published:** 2022-09-22

**Authors:** Han-Jun Ai, Matthias Beller, Xiao-Feng Wu

**Affiliations:** Dalian National Laboratory for Clean Energy, Dalian Institute of Chemical Physics, Chinese Academy of Sciences, China; Leibniz-Institut für Katalyse e.V., Germany; Leibniz-Institut für Katalyse e.V., Germany; Dalian National Laboratory for Clean Energy, Dalian Institute of Chemical Physics, Chinese Academy of Sciences, China; Leibniz-Institut für Katalyse e.V., Germany

The development of remote functionalization of alkenes has recently gained considerable attention as it offers the opportunity to directly transform a mixture of unrefined olefin isomers into a single valuable fine chemical by means of a chain-walking strategy [1–3]. Notably, in the chemical industry, a variety of such mixtures are available and less expensive compared with pure substrates. In addition, functionalization of olefinic mixtures offers possibilities for greener and more sustainable processes as purification steps are omitted. However, methods for selective remote functionalization of internal alkenes remain a challenge.

Recently, Professor Guosheng Liu's team at SIOC (Shanghai Institute of Organic Chemistry, Chinese Academy of Sciences) developed a novel catalytic remote hydrohalogenation of alkenes [4]. This protocol utilizes a specific palladium catalyst and provides easy access to primary alkyl chlorides and bromides with excellent linear selectivity under oxidative conditions (Fig. 1). The characteristics of this procedure include (i) employing suitable oxidants (NCS and Na-NMBI) to balance the reduction and oxidation steps; (ii) adding a small amount of H_2_O to activate hydrosilane and promote the formation of Pd^II^–H; (iii) using catalytic amount of triethylamine to facilitate the reduction of Pd^IV^–Cl intermediate to the desired alkyl chlorides. As shown in Fig. 1B, the activation energy for the oxidative halogenation of linear alkyl C_L_–Pd^II^ species (Path 3) is generally higher than that of branched alkyl C_B_–Pd^II^ species (Path 4) in the absence of ligand (ΔG_a_^‡^ > ΔG_b_^‡^). To overcome this challenge, the authors designed modified pyridinyl–oxazoline (Pyox) ligands **L1** and **L2**. The 6-substitued pyridyl group provided the driving force for chain-walking and the introduction of the hydroxyl group into oxazoline significantly accelerated the oxidative halogenation of C_L_–Pd^II^ species.

**Figure 1.**
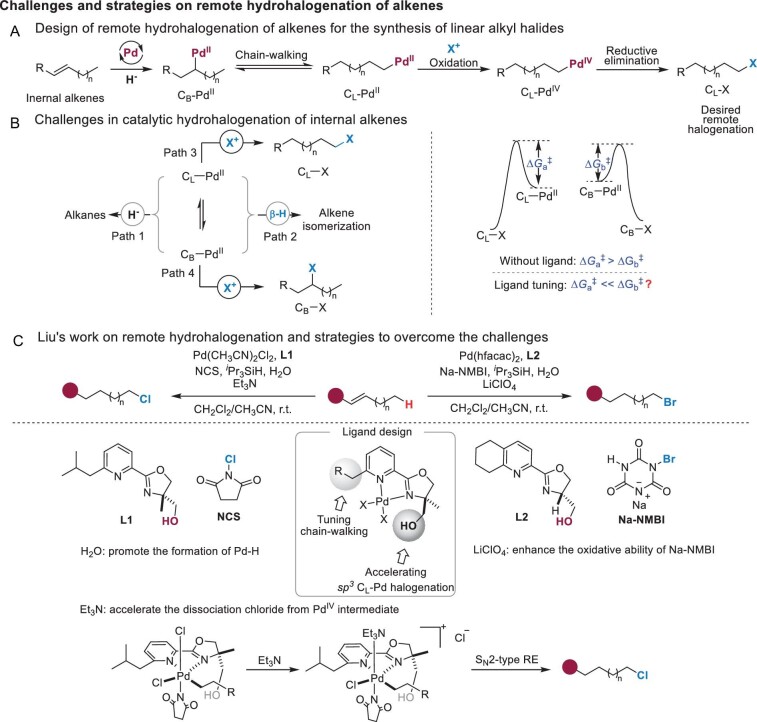

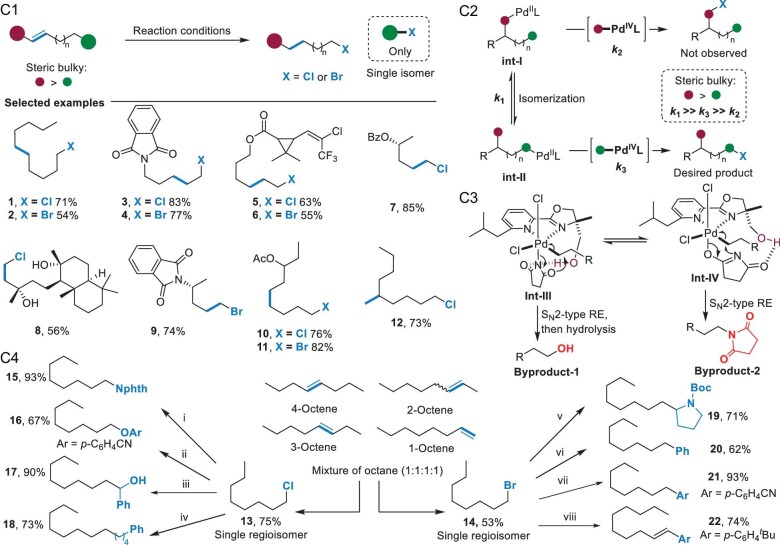
Challenge, strategy and selected results.

As this protocol proceeds under very mild conditions, a wide range of internal and terminal alkenes can be successfully transformed to primary alkyl halides in good yields (Fig. 1, C1, **1**–**12**). The scope also showed that the regioselectivity is mainly affected by steric hindrance, in which the sterically bulkier alkyl–Pd^II^ species **int-I** undergoes much slower oxidative halogenation than the less sterically hindered **int-II** (Fig. 1, C2). Furthermore, during their optimization process, the oxygenation (**byproduct-1**) and amination (**byproduct-2**) byproducts were also observed, which were derived from the intermediates **int-III** and **int-IV** (Fig. 1, C3). The remote hydrohalogenation of mixtures of octene isomers provided the single regioisomers **13** and **14** (Fig. 1, C4), which were further transformed into various other compounds (**15**–**22**). In summary, it is anticipated that this work will have a broad impact on the remote functionalization of internal alkenes and serve as an inspiration for new modes of such transformations.

## References

[1] Vasseur A , Bruffaerts J, Marek I. Nat Chem 2016; 8: 209–19. 10.1038/nchem.2445 26892551

[2] Sommer H , Juliá-Hernández F, Martin R et al. ACS Cent Sci 2018; 4: 153–65. 10.1021/acscentsci.8b00005 29532015 PMC5833012

[3] Bonfield HE , Valette D, Lindsay DM et al. Chem Eur J 2021; 27: 158–74. 10.1002/chem.202002849 32744766 PMC7821197

[4] Li X , Jin J, Chen P et al. Nat Chem 2022; 14: 425–32. 10.1038/s41557-021-00869-x 35102326

